# Prevalence of new onset erectile dysfunction among Damietta governorate men after contracting COVID-19

**DOI:** 10.1007/s11845-024-03610-y

**Published:** 2024-01-21

**Authors:** Mostafa A. Ahmed, Zakaria M. Obaid, Osama Hashem, Atef W. Elrifai, Mohamed L. Elsaie

**Affiliations:** 1 Department of Dermatology, Venereology and Andrology, Damietta Faculty of Medicine, Al-Azhar University, Damietta, Egypt; 2 Department of Pulmonology, Damietta, Faculty of Medicine, Al-Azhar University, Damietta, Egypt; 3 Department of Dermatology, Venereology and Andrology, Medical Research and Clinical Studies Institute, National Research Centre, Cairo, Egypt

**Keywords:** Androgen, COVID-19, Erectile dysfunction, Impotence, Testosterone

## Abstract

**Background:**

Several reports showed that sexual function was affected during and after the COVID-19 pandemic.

**Aims:**

The objective of the study was to demonstrate whether a realistic association exists between the COVID-19 pandemic and erectile dysfunction (ED) among a sampled Egyptian population in Damietta governorate.

**Methods:**

This cross-sectional study consisted of 330 adult males diagnosed with COVID-19 infection. They were categorized in three age groups (18–29, 30–39, and 40–50 years, respectively). COVID-19-confirmed cases were assessed by the Arabic version of the International Index of Erectile Function questionnaire (IIEF) scores.

**Results:**

The prevalence of ED according to the IIEF was 55.1%. The ED was categorized into mild ED which represents 25.8% of the patients, mild to moderate which represents 22.4% of the patients, moderate which represents 7% of the patients, and severe which represents 0% of the patients. We found a significant negative correlation between the age of the patients and the IIEF score. Also, we found an association between the severity of COVID-19 infection and the IIEF score.

**Conclusion:**

An association of new-onset ED in men who suffered COVID-19 infection was established. This may be due to virus-induced endothelial cell dysfunction; however, an underlying mechanism and causation have not yet been clearly elucidated. While it appears that COVID-19 infection may be a risk factor for ED, additional research is needed to establish causality.

## Background

An overwhelming health concern about cases suffering from acute respiratory distress was first discovered in Wuhan, Hubei Province, China, in December (2019). These cases turned out to be affected by coronavirus disease 2019 (COVID-19) with substantial casualties. The etiology of COVID-19 had been determined as a novel coronavirus, now known as severe acute respiratory syndrome coronavirus (SARS-CoV-2) [1].

Several reports showed that sexual function was affected among males and females during and after the pandemic [2–7]. Men’s complaints have included erectile dysfunction (ED) and decreased sexual satisfaction in comparison to a pre-COVID state [5]. Of note, many patients have reported a decrease in erectile function (EF), which was confirmed by a reduction of their International Index of Erectile Function (IIEF) assessment [8].

The high incidence of sexual dysfunction during the pandemic led to assumptions of biological underlying mechanisms such as hypogonadism, endothelial, and cardiopulmonary dysfunction were proposed [9].

End-organ damage seen with severe COVID-19 infections is thought to be a result of endothelial dysfunction, resulting both from direct infection and a systemic inflammatory response. Electron microscopy has demonstrated viral elements in the endothelial cells of affected organs including the penis [11].

Moreover; several other studies have shown that psychological disturbances, e.g., anxiety or depression, were the principal underlying etiology of sexual dysfunction during the pandemic [6–8].

The aim of the study was to demonstrate whether a realistic association exists between the COVID-19 pandemic and erectile dysfunction (ED) among a sampled Egyptian population in Damietta governorate.

## Methods

This cross-sectional study consisted of 330 adult males diagnosed with COVID-19 infection. They were categorized into the following age groups 18–29, 30–39, and 40–50 years. This categorization was chosen as it was used by similar studies and seems to be the most suitable in covering the targeted population and in producing clear statistical results. Patients were recruited from the post-COVID follow-up outpatient clinic at Al-Azhar University Hospital, Damietta over a period of 6 months from March 2022 to August 2022. Our study followed the Helsinki Declaration principals. Ethical approval was obtained from the institutional review board of Damietta Faculty of Medicine (Al-Azhar University). Written informed consent was obtained from every patient at the recruitment. Inclusions included males aged 18–50 years of age who are sexually active and who were diagnosed with COVID-19 infection (only those who finished the management protocol and had been discharged at least 2 weeks prior to joining the study). Subjects with confirmed erectile dysfunction (ED) due to known causes were excluded. All patients who were receiving any medications that could cause ED including anti-anxiety agents, antidepressants, antipsychotics, or mood stabilizers were not recruited. Moreover; all subjects with endocrinal diseases, neurological diseases, prostatic diseases, pelvic trauma or spinal cord injury, Peyronie’s disease or curvature, alcoholics, metabolic syndrome, and chronic illnesses (diabetes mellitus, hypertension, liver disease, renal failure, and cardiovascular disease) were also excluded from the study.

## Data collection

All patients were subjected to the following.

1. Full history taking (medical and surgical), marital status, occupation, sexual activity, and ED
2. COVID-19 confirmation by using the reverse transcription polymerase chain reaction (RT-PCR) test of pharyngeal and nasal swabs. The severity of COVID-19 was determined by following the guidelines stated by Zhu et al. [12].
3. Clinical examination and evaluation of potency were done by the validated Arabic version of the International Index of Erectile Function (IIEF) [13]. The IIEF Questionnaire was developed to address the need for a self-report measure of both erectile function and sexual function that can be given under guidance of a clinician. The IIEF Questionnaire presents the quality of male sexual function in terms of five domain scores: erectile function, orgasmic function, sexual desire, intercourse satisfaction, and overall satisfaction. This questionnaire consists of only five questions, and each IIEF-5 item is scored on a five-point ordinal scale where lower values represent poorer sexual function [10]. Thus, a response of 0 for a question was considered the least functional, whereas a response of 5 was considered the most functional. The possible scores for the IIEF5 range from 1 to 25 (one question has scores of 1–5), and a score above 21 was considered as normal erectile function and at or below this cutoff, ED. According to this scale, ED is classified into four categories based on IIEF-5 scores: severe (1–7), moderate (8–11), mild to moderate (12–16), mild (17–21), and no ED (22–25) [14].

The data entry and statistical analyses were performed using SPSS (Statistical Package of Social Sciences) version 26 (SPSS Inc., Chicago, IL, USA). Continuous normally distributed data were expressed in mean and standard deviation. The quantitative data were examined by Kolmogorov–Smirnov test for normality of data. Kruskal–Wallis test was used for continuous not normally distributed data. Categorical data were described as numbers and percentages and were analyzed using the Chi-square test. Statistical significance was considered when probability (*P*) value is less than or equal to 0.05.

## Results

This study included 330 patients diagnosed with COVID-19 infection. The mean age of the patients was 35.7 ± 7.34 years with a range of 25–49. According to the severity of infection, 54.2% of the patients were mild, 34.2% were moderate, 7.8% were severe, and 3.6% were critical. In terms of the duration between the infection and the occurrence of ED, the mean duration was 6.7 ± 3 months (Tables 1 and 2).

**Table 1:** Demographics and the clinical characteristics of the studied patients

**Variables**	**Mean ± SD or** ***N* (%)**
**Age**	
**Mean ± SD**	35.7 ± 7.34
**Range**	25–49
**COVID-19 severity. *N* (%) (*N* = 330)**	
**Mild**	179 (54.2%)
**Moderate**	113 (34.2%)
**Severe**	26 (7.8%)
**Critical**	12 (3.6%)
**Time between infection and ED**	
**Mean ± SD**	6.7 ± 3
**Median (IQR)**	6 (5–9)
**Range**	1–15

*ED* erectile dysfunction

**Table 2:** International Index of Erectile Function different questionnaire scores

**Scores**	**Very low**	**Low**	**Moderate**	**High**	**Very high**
**Q1. Confidence level**	0 (0%)	29 (8.8%)	86 (26.1%)	124 (37.6%)	91 (27.6%)

**Scores**	**Almost**	**Few times**	**Some times**	**Most times**	**Always**
**Q2. Ability of erection to penetrate. *N* (%)**	0 (0%)	34 (10.3%)	76 (23%)	101 (30%)	119 (36.1%)
**Q3. Ability to maintain erection after penetration. *N* (%)**	18 (5.5%)	11 (3.3%)	87 (26.4%)	95 (28.8%)	119 (36.1%)
**Q4. Satisfaction with intercourse**	0 (0%)	30 (9.1%)	91 (27.6%)	71 (21.5%)	138 (41.8%)

**Scores**	**Extremely difficult**	**Very difficult**	**Difficult**	**Slightly difficult**	**Not difficult**
**Q5. Difficulty to maintain erection. *N* (%)**	0 (0%)	36 (10.9%)	82 (24.8%)	115 (34.8%)	97(29.4%)

Erectile function of the patients was assessed by the IIEF score, which consisted of 5 questions. The mean IIEF score was 19.45 ± 4.36 with a range of 10–25. Based on this score, the patients were divided into mild ED (22.8%), mild to moderate ED (22.4%), and moderate ED (7%). Severe ED was not reported in our study (Tables 3, 4, and 5).

**Table 3:** Total International Index of Erectile Function scores and classification

**Variables**	**Mean ± SD or *N* (%)**
**Total IIEF score**	
**Mean ± SD**	19.45 ± 4.36
**Range**	10–25
**Total IIEF categories**	
**No**	148 (44.8%)
**Mild**	85 (25.8%)
**Mild to moderate**	74 (22.4%)
**Moderate**	23 (7%)
**Severe**	0 (0%)

*IIEF* International Index of Erectile Function

**Table 4:** IIEF score according to COVID-19 severity

**Variables**	**Mild COVID-19 infection**	**Moderate COVID-19 infection**	**Severe COVID-19 infection**	**Critical COVID-19 infection**	*P* value*^a^
**IIEF score**	23 (20–24)	16 (14–19)	16 (11–19)	18.5 (14.5–22.7)	0.001*

*IIEF* International Index of Erectile Function

*Significant *P* value

^a^Kruskal–Wallis test

**Table 5:** Association between the IIEF category and the COVID-19 severity

**Variables**	**Mild COVID-19 infection (*N* = 179)**	**Moderate COVID-19 infection (*N* = 113)**	**Severe COVID-19 infection (*N* = 26)**	**Critical COVID-19 infection (*N* = 12)**	***P* value**
**No**	125 (69.8%)	20 (17.7%)	3 (11.5%)	0 (0%)	0.001*^a^
**Mild**	42 (23.5%)	35 (31%)	7 (26.9%)	1 (8.3%)	
**Mild to moderate**	9 (5%)	47 (41.6%)	9 (34.6%)	9 (75%)	
**Moderate**	3 (1.7%)	11 (9.7%)	7 (26.9%)	2 (16.7%)	
**Severe**	0 (0%)	0 (0%)	0 (0%)	0 (0%)	

*IIEF* International Index of Erectile Function

*Significant *P* value. % percentage per column

^a^Chi-square test

By comparing the different degrees of COVID-19 infection according to the IIEF score, the IIEF score was significantly lower in patients with moderate to severe and severe COVID-19 infection (*P* value = 0.001) (Table 6).

**Table 6:** Association between the IIEF category and age groups

**Variables**	**20–29 years (*N* = 85)**	**30–39 years (*N* = 128)**	**40–50 years (*N* = 117)**	***P* value**
**No**	41 (48.2%)	65 (50.8%)	42 (35.9%)	0.001*^a^
**Mild**	29 (34.1%)	27 (21.1%)	29 (24.8%)	
**Mild to moderate**	9 (10.6%)	24 (18.8%)	41 (35%)	
**Moderate**	6 (7.1%)	12 (9.4%)	5 (4.3%)	
**Severe**	0 (0%)	0 (0%)	0 (0%)	

*Significant *P* value. % percentage per column

^a^Chi-square test

Correlation analysis revealed a statistically significant correlation between the IIEF score and the severity of infection, in which most of the patients with ED were mild and moderate COVID-19 infection (*P* = 0.001) (Table 7).

**Table 7:** Correlation between IIEF score and category and different study variables

**Variables**	**IIEF score**	
	***r***	***P* value**
**Age**	− 0.1	0.04*
**COVID-19 severity**	− 0.5	0.001*
**Duration of infection**	0.1	0.09

*IIEF* International Index of Erectile Function

**Fig. 1:**
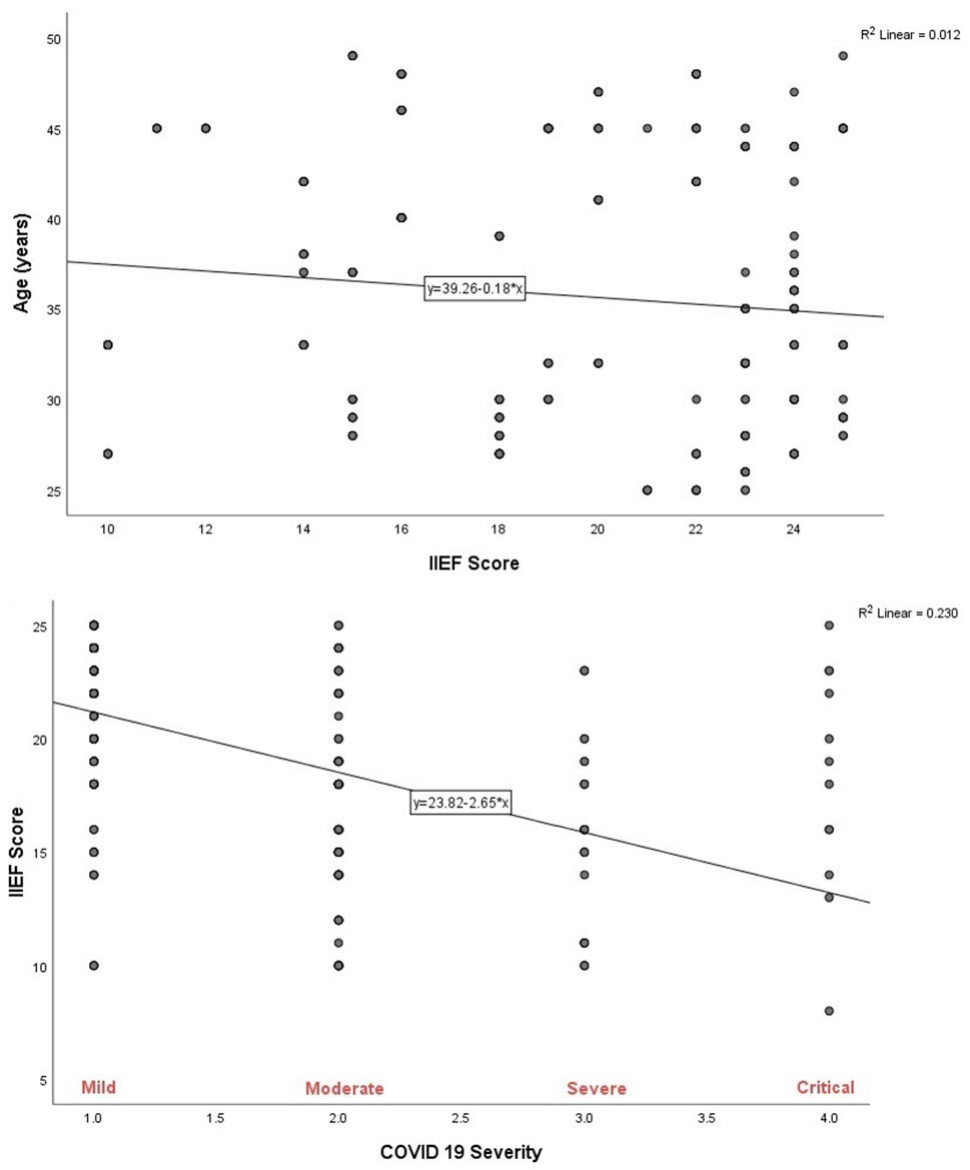
Correlation of International Index of Erectile Function (IIEF) to age (above) and COVID-19 severity (below)

In the present study, most of the ED cases were in the age category of 40–50 years; however, most of the normal EF cases were in the age category of 30–39 years. Also, a statistically significant association was found between the degree of ED and the age category in which most of the mild to moderate and moderate ED were in the age category of 40–50 years (*P* value = 0.001) (Table 7 and Fig. 1).

## Discussion

Previously, COVID-19 has been shown to have harmful effects on various organs throughout the body, including the heart, kidneys, and vascular system [15]. Our current understanding of COVID-19 and its effect on the penile tissue and subsequent erectile function remains unclear. The importance of identifying the virus’ short and long-term effects on male reproductive organs is potentiated by a recent study observing COVID-19 particles in penile tissue through transmission electron microscopy and hematoxylin and eosin (H&E) staining [11]. As COVID-19 causes widespread endothelial dysfunction, and erectile function is dependent on a functional endothelium and proper vascular flow, it is hypothesized that a COVID-19 infection may be associated with new onset erectile dysfunction [16].

In this study, the association between males with COVID-19 infection and erectile dysfunction was investigated, and the prevalence of ED in COVID-19 patients was 55.1%, which is affirmative of another study that assessed the risk of ED in men with COVID-19 in the United States (US) and reported that COVID-19 diagnosis was significantly associated with ED [17].

Endothelial penile dysfunction in humans was reported following COVID-19 infection resulting in ED [18]. In addition to endothelial damage, there is a lack of clear understanding of the exact causes of ED among COVID-19-infected males; however, a systemic review found that stress, anxiety, and depression are among psychological factors that can be considered as possible causes of sexual dysfunction in COVID-19 patients [19]. Moreover, financial constraints and social restrictions experienced during the pandemic were among aggravating causes of sexual dysfunction among males [20–24].

Another important cause for sexual dysfunction is the fear and perception of contracting and/or spreading COVID-19 through sexual intercourse [25–27].

Moreover, it was reported that the men at highest risk of developing serious complications secondary to COVID‐19 also have a higher risk of ED. This group consisted of old-aged individuals, diabetics, subjects with cardiovascular disease, overweight/obese individuals, and those with multiple co-morbidities [28].

Studies investigating the pathogenesis of SARS‐CoV‐2 have revealed that the virus binds to the ACE2 receptor and enters the cell through the type 2 transmembrane serine protease 2 (TMPRSS2) enzyme found in the host cells [29]. ACE2 and TMPRSS2 are expressed in host target cells, particularly alveolar epithelial type II cells. They are also expressed in the digestive, cardiovascular and urinary systems, and testicular tissue [30].

In light of this information, Zhu et al. constructed a risk map of COVID‐19 using RNA sequencing datasets to evaluate the expression of the ACE2 receptor in various tissues and cells. According to this risk map, in addition to the respiratory system; cardiovascular, renal, gastrointestinal, and urogenital systems were also shown to be potentially vulnerable to the virus due to ACE2 expression [12].

Moreover, SARS-CoV-2 gonadal involvement was shown to increase serum prolactin levels and lowering testosterone levels via pituitary gland suppression, further adding to erectile function deterioration [31–33].

Kresch et al. examined patients who had symptomatic COVID‐19 infection and subsequently developed severe ED. The authors took tissue samples at the time of penile prosthesis implantation, and as a result of the histopathological examination, they stated that the systemic effect of COVID‐19 or widespread endothelial damage caused by the virus directly affecting the vascular endothelium could have a negative impact on penile vascular flow and lead to the deterioration of the erectile function [11].

In another study, Sivritepe et al. evaluated changes in the IIEF scores of 80 sexually active male patients hospitalized due to symptomatic COVID‐19 infection. According to the evaluations they performed during hospitalization and at the third month after discharge, there was a positive correlation between ED and the interleukin‐6 level. The authors attributed this correlation to the vascular consequences of possible interleukin‐6‐related inflammation [34].

In the present study, we found a significant negative correlation between the age of the patients and the IIEF score. This came in agreement with a cohort study that reported erectile dysfunction is strongly age-related with an approximately 2 to threefold increase in the prevalence of moderate-to-severe ED between the ages of 40 and 70 years [35]. A variety of medical, psychological, and hormonal statuses have been implicated in the etiology of ED. Testosterone is one of these factors. A relationship between aging and serum androgen levels was investigated in older men. The results are still controversial [36].

In the current study, we found an association between the severity of COVID-19 infection and the IIEF score. This is in agreement with Bary et al., who explained this correlation by a broad range of mechanisms including local direct and indirect effects on endothelial cells, secondary hypogonadism, and endocrine dysfunction as well as psychosocial factors [37].

In the current cohort, the deterioration in the erectile functions of moderate and severe patient groups had significantly lower IIEF score and higher prevalence of ED. In order to achieve a good erection, adequate blood flow to the penis must be provided. It is clear that this requires a well‐functioning cardiopulmonary system. COVID‐19 targets vital organs, such as the heart and lungs, it may take these systems up to 6 months to recover their functions after the disease, and there may sometimes be irreversible damage [38].

It is reported that the main cause of ED in lung patients is decreased functional capacity due to hypoxemia [38]. In a multicenter prospective study, Sonnweber et al. reported that a significant percentage of post‐COVID‐19 patients presented with radiological pulmonary abnormalities and pulmonary dysfunction 100 days after diagnosis. However, the authors also noted a significant improvement in symptoms and cardiopulmonary status over time [39].

In a study examining the possible mechanisms of the long‐term effects of COVID‐19 on sexual health, it is generally stated that the long‐term effects of the disease are proportional to the presence and severity of the accompanying non‐communicable diseases [40]. Since we examined the short‐term results of our patients, we consider that the deterioration in IIEF‐5 scores may have been related to impaired exercise capacity and cardiopulmonary function associated with COVID‐19. There is a need for further studies examining long‐term outcomes in order to validate this hypothesis.

Our sample size was considerable, and all participants were confirmed COVID-19 diagnosis. However, several limitations exist to the current work with the absence of comparison group and pre-existed erectile function status, the assumption of whether COVID-19 was the cause of ED could not be concluded and the confounding bias from social situations such as health policy during pandemic and change in sexual habit might affect the erectile function.

## Conclusion

In conclusion, we reported an association of new-onset ED in men who had COVID-19 infection. This may be due to virus-induced endothelial cell dysfunction; however, an underlying mechanism and causation have not yet been clearly elucidated. While it appears that COVID-19 infection may be a risk factor for ED, additional research is needed to establish causality. The study reported the prevalence of ED to be higher in male patients older than 40 years old and that erectile function was significantly affected by the severity of COVID-19 infection. Future studies are needed to determine the predictive factor of ED in COVID-19 patients and appropriate early interventions.

## Data Availability

The data that support the findings of this study are available from the corresponding author upon reasonable request.

## Abbreviations

ED: Erectile dysfunction
IIEF: International Index of Erectile Function
ACE2: Angiotensin-converting enzyme 2
COVID-19: Coronavirus disease 2019
EF: Erectile function
MERS-CoV: Middle East respiratory syndrome coronavirus
SARS-CoV-2: Severe acute respiratory syndrome coronavirus 2
